# Cnidaria-Inspired Morphing Mechanism for Underwater Robot: A Soft Tectonics Approach

**DOI:** 10.3390/s25216780

**Published:** 2025-11-05

**Authors:** Yin Yu

**Affiliations:** 1 School of Art and Design, College of Professional Studies and Fine Arts, San Diego State University, San Diego, CA 92083, USA; yyu2@sdsu.edu; 2 Center for Multisensory Architectural Research, San Diego State University, San Diego, CA 92083, USA

**Keywords:** soft tectonics, elastic structure, soft mechanism, underwater robot, morphologies, soft structure, soft robotics, cnidaria, sea anemone

## Abstract

Soft robots demonstrate great potential for underwater exploration, particularly in tasks such as locomotion and biological sampling in fragile marine habitats. However, developing new forms of interaction with underwater life remains a challenge due to inadequate soft mechanisms for studying the behavior of marine invertebrates. We present a 7-cm in diameter anemone robot (“Soromone”) capable of performing biological sea anemones’ wiggling behavior under the water. Inspired by the body forms of adult cnidaria, we developed a morphing mechanism that serves as both structure and actuator for the Soromone’s behavior using a soft tectonics approach—a multistep, multiscale, heterogeneous soft material fabrication technique. As an actuator, the morphing mechanism can precisely control the Soromone via a fluid system; as a structure, it can reinstate the Soromone’s original shape by incorporating various degrees of stiffness or softness into a single piece of material during fabrication. Our study demonstrates the advantages of applying a Soromone under water, including increasing water flow for enhanced nutrient uptake, waste removal, and gas exchange. This cnidaria-inspired soft robot could potentially be adapted for interaction with coral reef ecosystems by providing a safe environment for diverse species. Future soft robotics design paradigms based on a soft tectonics approach could expand the variability and applicability of soft robots for underwater exploration and habitation.

## 1. Introduction

Scientists predict the future will be a world where robots become as common as cars or phones, where everyone could have a personal robot by 2040 [1]. Due to safety and compliance considerations, it is highly unlikely that such personal robots would be made only of rigid materials like our phones or computers. Unlike such conventional rigid structures, soft robotics is an emerging field in robotics that has seen an exponential growth of interest both in the academy and in industry [2]. Baymax, a popular culture soft robot from the 2014 Disney film Big Hero 6, is a fictional inflatable vinyl robot that serves as a personal health care provider and companion. In the real world, thanks to their significant flexibility, deformability, and adaptability, soft robots offer unique opportunities in areas in which conventional robots are not viable, such as the therapeutic robot Paro for older adults [3], non-invasive surgical procedures [4], drug delivery [5], artificial organs [6], and underwater biological sampling [7]. Among the variety of potential future applications, soft robotics could achieve breakthrough developments in underwater exploration due to their flexibility and adaptability. There is a clear need to design future underwater soft robots that perform physical tasks and, more importantly, co-live responsibly with other marine animals in complex, built and natural environments.

Despite obvious advantages and promising uses, soft robotics as a new multidisciplinary field faces many challenges [8]. One of them is how to evolve soft robots’ behaviors, which to date have mainly been explored for carrying out conventional tasks. Recent reviews have classified soft robot operations as “gripper”, such as sampling, manipulating; and “propulsion/locomotion”, such as swimming, crawling, digging, walking, running, jumping, climbing, flying, and multimodal [9]. However, soft robot behavior can expand beyond gripping and propulsion. In addressing this gap, we note that existing soft robots for underwater exploration have limited capabilities. Following Katzschmann et al. observations from their experiments with underwater soft robotic fish, they suggest that future studies should include studying the behavior of marine life over long periods of time without human interference with the natural environment and observing whether the robot may influence the behavior of marine life [10].

In the field of soft robotics, cnidarians offer a great source of inspiration due to their soft, deformable tissues, flexible movements, and adaptive behaviors in aquatic environments. As a phylum of aquatic invertebrate animals, Cnidaria comprises over 11,000 species including jellyfish, sea anemones, corals. In our study, we aim to investigate soft robot behavior from the perspective of a symbiotic relationship. We focus on sea anemone, a group of predatory marine invertebrate animals that plays a crucial role in providing microhabitats for diverse fauna in tropical coastal habitats such as coral reefs [11,12]. Coral reefs form marine biodiversity hotspots of enormous ecological, economic, and aesthetic importance [13]. For example, the tropical coral reef, a great biodiversity source, occupies only 0.1% of the ocean, but it serves as the habitat for 25% of all known marine species (although many more species are likely to be discovered in them) [14]. The study of the morphology of aquatic creatures, particularly of cnidarians, is not only ecologically significant but also urgent for advancing soft robotics-based research.

Underwater robotics research on zoomorphic types includes fish-like swimming robots [10], octopus-like robots [15,16], jellyfish-like robots [17], flatfish robots [18], and sea anemone-inspired robots [19,20,21,22]. Previous third-party projects cited above demonstrate the great potential of marine animal-inspired soft robotics research. However, existing design methods often avoid the challenge of mimicking the morphology, movement, and behavior of marine invertebrates, such as sea anemones—whose unique body plan, tentacle dynamics, and feeding strategies provide a promising yet underexplored model for developing new soft robotic mechanisms. As those researchers observed, the complexity of such robotic systems, due to their unconventional components from materials to actuation, makes it hard to use currently known design and simulation tools to build soft robots [23]. The driving motivation of this study is not only mimicking sea anemone forms but also offering innovative design solutions and methods to improve and update current approaches.

In this paper, we introduce our soft robot anemone, Soromone. Section 2 presents an overview of our cnidaria-inspired biomimicry design and describes the soft tectonics methodology. Then, in Section 3, we study and validate two types of actuations and present our experiments. Finally, in Section 4, the results of the experiments are presented, followed by a brief conclusion.

Our study aims to develop a novel soft robot for public exhibition that advances knowledge of ethorobotics, an emerging discipline that brings together the fields of animal behavior and robotics [24]. To the best of our knowledge, ours is the first publicly oriented soft robot anemone used at an art gallery for a general audience. Our experiment contributes to society by emulating the performance of the biological systems of sea anemones in wet environments. The main contributions of this work can be summarized as follows:

(1) A state of the art of soft robot that mimics a complex morphology;
(2) A state of knowledge via a new design guideline—soft tectonics;
(3) A demonstrated experimental result with precise control of inflation and deflation, laying the groundwork for future studies;
(4) The validation of our proposed method in a small-scale water tank.

## 2. Materials and Methods

**Figure 1:**
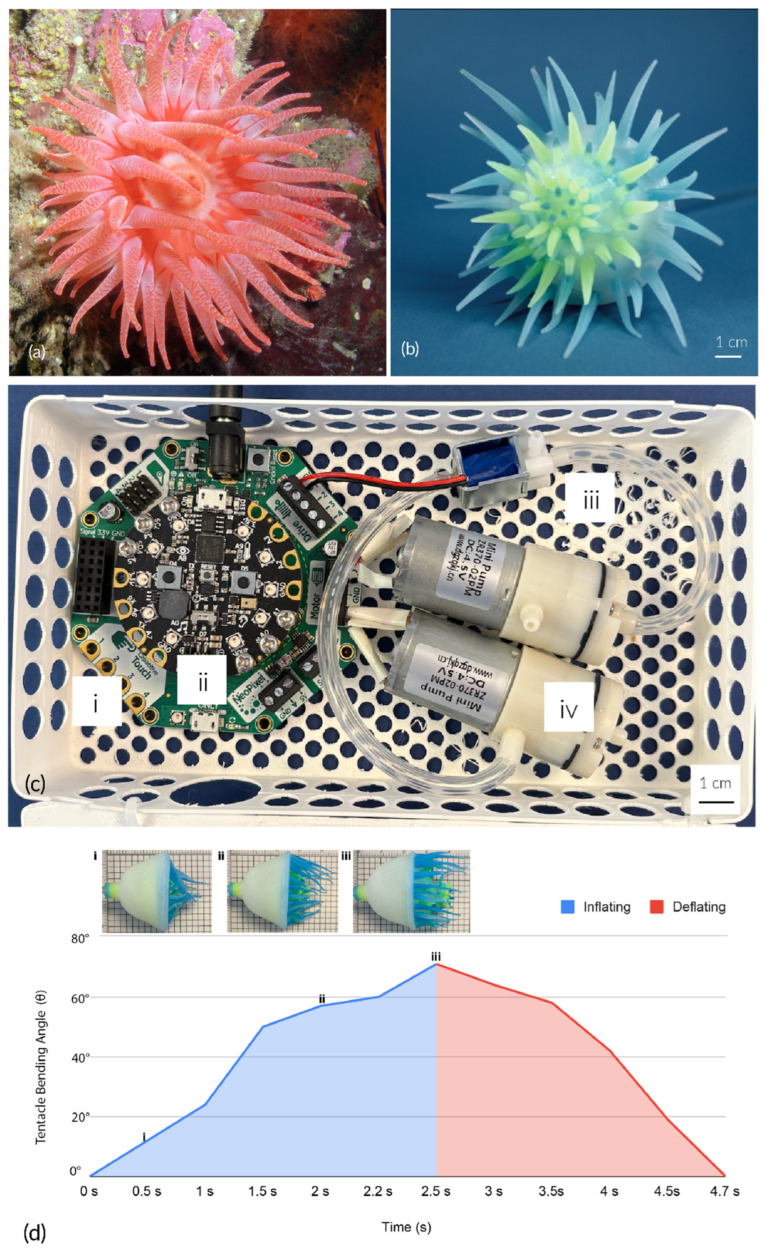
Overview of Soromone: (**a**) A crimson anemone (source: https://en.wikipedia.org/wiki/Cribrinopsis_fernaldi, accessed on 26 October 2025); (**b**) A Soromone in a morphed state; (**c**) A pneumatic actuation computer control system includes (i) an Adafruit Crickit with an external power supply connected to (ii) an Adafruit Circuit Playground Express, (iii) one air valve wired to the drive output on the Crickit, and (iv) two air pumps wired to the DC motor control outputs on the Crickit; (**d**) A full cycle of pneumatic actuation controlled by the computer system takes 2.5 s to inflate and 2.2 s to deflate, and (i)–(iii) illustrate Soromone at 0.5 s, 2.0 s, and 2.5 s, respectively.

Soromone is a soft actuator that can perform a biological sea anemone’s wiggling behavior and feeding movement either in air or submerged underwater (Figure 1). We introduce a novel soft robotics design methodology—Soft Tectonics, which investigates the relationship between a three-dimensional elastic structure, soft material, and biological morphologies. We achieve the desired behavior and document our manufacturing process. We realize a morphing mechanism that can actuate pneumatically or hydraulically.

In this section, we first present an overview of our project–Soromone. Then, we introduce our design method of soft tectonics which was then used in the design and manufacturing of the Soromone. Following that, we give a detailed description of cnidaria-inspired biomimicry design of the Soromone.

### 2.1. Soromone: Material Overview

**Figure 2:**
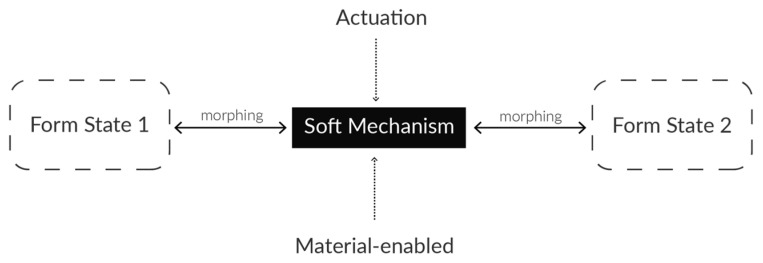
Elements of soft tectonics for soft robot.

With an emphasis on bioinspired motion mimicry, one of our primary concerns is to design a robot structure that performs as flexibly and organically as possible. Following a soft tectonics design principle, as illustrated in Figure 2, we propose a conceptual design inspired by sea anemones (Figure 1a). The conceived robot consists of two main components: a soft robotic body and a pneumatic actuation system.

#### 2.1.1. Soft Actuator

A Soromone is a soft actuator made entirely of soft silicone material (Smooth-On Inc., Dragon Skin FX-Pro, East Texas, PA, USA). We use the pigment color Ignite’s blue and yellow (Smooth-On Inc., Fluorescent Color Pigments, Blue PMS 300C, and Yellow PMS 809C, East Texas, PA, USA) to mimic deep-sea fluorescent organisms. Under no actuation, the Soromone has a diameter of 7 cm, and its body length is 6 cm. When the soft robot is actuated, the tentacles will stretch outward and the mouth will become visible. The length of the robot can reach 12 cm. Figure 1b shows one Soromone with tentacles extended under actuation.

#### 2.1.2. Control System

A Soromone can be actuated by a fluid control system. At this stage in our study, we have manually tested controlled hydraulic and pneumatic approaches using a syringe. We have also tested a computer controlled pneumatic approach through electronics. In this section, we detail the material we used for our pneumatic-based computer control system as shown in Figure 1c.

The pneumatic actuation control system (Figure 1c) is housed in a custom 3D-printed porous box (18 cm by 10 cm by 4 cm) for easy mounting and cable arrangement. Inside the box, the system includes:

(i) An Adafruit CRICKIT (Adafruit Industries, Brooklyn, NY, USA);
(ii) An Adafruit Circuit Playground Express (Adafruit Industries, Brooklyn, NY, USA);
(iii) A 6V air valve (ZhiRongHuaGuan, Dongwan, China);
(iv) Two 4.5 V 2.5 LPM air pump DC motors (ZhiRongHuaGuan, Dongwan, China);
(v) An 5V 2A AC/DC power adapter (Adafruit Industries, Brooklyn, NY, USA).

See these materials labeled as i to iv in Figure 1c.

#### 2.1.3. Experiment Setup

For our experiments in Section 3.2, we used a glass water tank measuring 25 cm × 25 cm × 25 cm (AquareCraft, Ningbo Rui Mu Shui Yi Technology Co., Ltd., Ningbo, China), a 60 mL syringe, and California tap water. We also prepared the fluorescein dye by mixing fluorescein sodium (Carolina Biological Supply Company, Burlington, NC, USA) with filtered tap water.

### 2.2. Soft Tectonics: A Method for Soft Robotics Design

#### 2.2.1. Traditional Biomimicry Design and Fabrication

Soft robots and conventional rigid robots use different mechanisms to enable actuation. Thus, conventional actuation mechanism design cannot simply be adapted to soft robotics. The actuation mechanism of soft robotics requires a different approach. The design of soft robotics is deeply grounded in bioinspired, biomimetic, and biohybrid approaches because living organisms are mostly composed of soft tissues. Another grand challenge in robotics is bioinspired and biohybrid robots that translate fundamental biological principles into engineering design rules or integrating living components into synthetic structures to create machines that perform like natural systems [8]. With various mechanisms, structures, and motion performance, soft actuators have been key in soft robotics in recent decades. For the scope of this study, we focus on soft fluidic elastomer devices based on their fast response time, cost efficiency, and highly biomimetic movement. Most current soft actuators can be classified into three types of morphologies: ribbed segment, cylindrical segment, and pleated segment [25]. These morphologies can perform different types of motion, such as bending, twisting, and contracting/elongating [26]. Common approaches for manufacturing soft actuators can be categorized into three methods: lamination casting, retractable pin casting, and lost wax casting [25]. Despite increasing publications about soft robotics, most soft fluid actuators still fall into the limited morphological categories due to a lack of new design principles. Our motivation is to invent a new actuation behavior through a novel design principle and a new fabrication method that could contribute to the soft robotic field and beyond.

#### 2.2.2. Soft Tectonics Design Principle for Soft Robotics

In architecture, tectonics originate intrinsically from the materiality of the architectural form and a materializing process called construction [27]. We propose to integrate soft tectonics [28] derived from modern architectural design theory, as it comprises the expressive integration of material, structure, and form in construction, which reflects evolution. The soft robotics-based soft tectonics is a design methodology that integrates soft material composition, elastic structural morphologies, and actuation principles into expressive forms and functional behaviors through soft mechanisms. Figure 2 illustrates the elements of soft tectonics design principle.

#### 2.2.3. Manufacturing Method

The manufacturing method of soft tectonics is based on the elements of the design principles from Section 2.2.2. The Soromones we build use the 3D printed molds casting method with a soft tectonics approach. We highlight some distinct characteristics:

- Biological-expression oriented

Traditional robotic manufacturing prioritizes the task or expected outcome. Consequently, many design processes begin from the outside and work inward: the robot is shaped to resemble an animal externally (e.g., a fish-like body form), while its interior is filled with rigid components, and joints that bear little resemblance to biological anatomy. In contrast, soft tectonics invert this logic by prioritizing biological expression and evolutionary logic. The design and manufacturing process begins from the inside and works outwards, rooting structure and actuation in principles inspired by real biological systems.

- Material-driven fabrication

Soft materials can deform in all directions, offering countless possibilities. The manufacturing process should therefore support this materiality by enabling customized methods that generate diverse outcomes for different functions and needs. In other words, the manufacturing process should be driven by the properties of the material itself. For example, a common challenge in soft robotics fabrication is the formation of air bubbles during elastomer casting, which results in reduced quality and performance [29]. This issue arises because traditional manufacturing methods focus on containing the material inside the mold, rather than allowing it to flow naturally. By contrast, if the process is designed to accommodate the natural flow of the material, trapped air can escape more easily. As an example, we adopt this material-driven technique in tentacle fabrication by adding a small escape channel, which allows air to flow out during casting and thus achieves consistent and reliable results.

- Geometry

Conventional robotic manufacturing has a long history of industrial standardization, where parts and joints are designed according to established norms. In rigid robots, mechanisms are defined as systems of rigid bodies connected by joints or linkages to achieve specific motions or to force transmission [30]. In contrast, the mechanisms of soft robotics are fundamentally different. The equivalent of “nuts and bolts” in soft systems requires an entirely new geometric language of morphing mechanisms. Consequently, new fabrication approaches and libraries of soft geometries are needed to support the design and assembly of these mechanisms. Researchers have also noted that manufacturing complex internal structures, volumetric cavities, and undercuts remain a major challenge [20]. Therefore, the manufacturing process must evolve to accommodate the unique geometries demanded by soft mechanisms. With the advancement of 3D digital modeling technologies and the conceptual framework of architectural tectonics, the development of bio-inspired geometric solutions for soft mechanisms is not a matter of possibility, but of time.

- Integration

Traditional manufacturing processes rely on the assembly of discrete parts, joints, and elements. In contrast, soft robotic manufacturing emphasizes seamless integration, embedding structure, actuation, and sensing into a unified body. Soft robots aim to mimic animal morphologies and behaviors that have evolved over thousands of years. Advancing soft robotic manufacturing therefore requires multidisciplinary, multiscale, and multistep approaches that combine diverse materials and fabrication techniques to ensure that form and function are inseparably linked.

### 2.3. Design and Manufacturing of the Soromone: An Example of Soft Tectonics Approach

#### 2.3.1. The Design of Soromone

Cnidarians have two distinct body forms: the medusa (e.g., jellyfish) and the polyp (e.g., sea anemone) [31]. The design of the Soromone draws inspiration from the body of the medusa and the tentacles of the polyp—an optimized combination of these two distinct body forms. A medusa’s body is diploblastic—consisting of endoderm and ectoderm—and separated by mesoglea, a variably thick layer that reinforces the medusa’s structure [32]. Arrays of tentacles are a unifying feature of cnidaria, with diverse species featuring distinct arrangements, morphologies, and numbers of tentacles. In the typical polyp bauplan, tentacles are extensions of the diploblastic body, forming appendages that feed, defend, and expand the surface area of the gastric cavity [33]. The radial symmetry of cnidarians’ body parts is the inspiration behind the design and manufacturing of the Soromones in this study.

**Figure 3:**
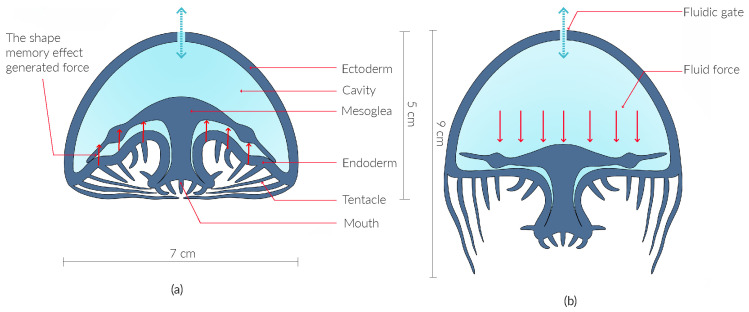
Design illustration of the Soromone: (**a**) depicts the Soromone in its unactuated state, with the mouth closed after deflation. During deflation, fluid comes out from the internal cavity, and the mesoglea is pushed upward due to its shape-memory characteristics as illustrated the up red arrow; (**b**) shows the Soromone in its actuated state, with the mouth open. In this phase, fluid enters the cavity and pushes the mesoglea downward as illustrated the down red arrow. The expansion of the cavity is schematically represented.

The body of a Soromone consists of three elements: the ectoderm, mesoglea, and endoderm. The ectoderm forms the bell-shaped exterior wall, enclosing and supporting all components of the Soromone. The mesoglea serves as the middle morphing mechanism, providing structural support while enabling deformation and restoring the body to its original shape. The endoderm forms a flower cup-shaped interior wall and exhibits the most dramatic shape changes. When assembled, these three layers create an internal cavity and the mouth, as illustrated in Figure 3. Together, they form the body of a Soromone, which functions as the robot’s soft actuator.

While the ectoderm and endoderm maintain a uniform wall thickness of 2 mm, the mesoglea requires a variable stiffness design to fulfill its function. Variations in material thickness and curvature are distributed across the mesoglea to optimize performance. It must balance flexibility for deformation with sufficient resistance to ensure reliable shape recovery. Figure 3 illustrates the forces applied to the internal elastic structure and its response once the force is released.

**Figure 4:**
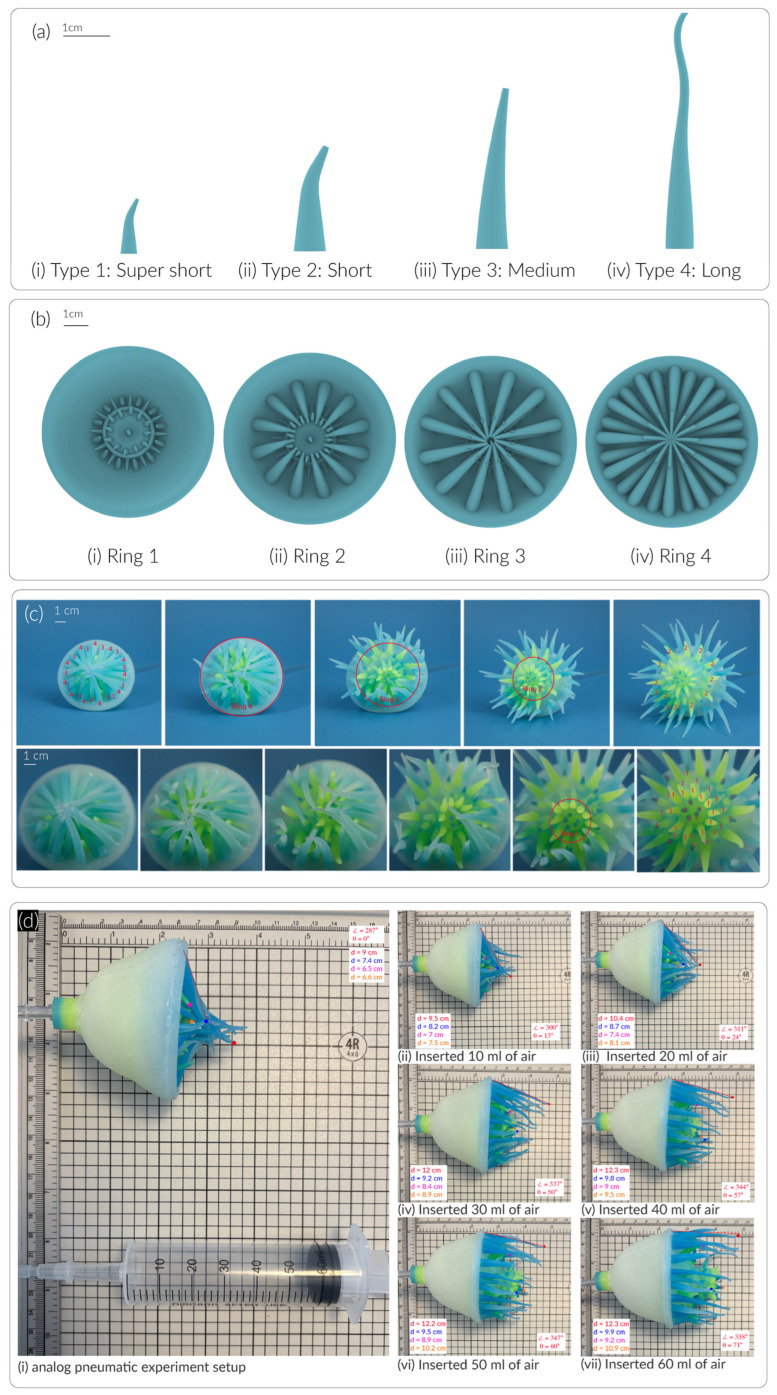
The tentacles of a Soromone and its performance in air. (**a**) Digital model of four types/sizes of tentacles; (**b**) The arrangement of the tentacles in a digital model; (**c**) A sequence of front views of a Soromone’s tentacles under pneumatic actuation in air with annotation tentacle Type I–IV as 1–4; (**d**) A sequence of side views showing Soromone’s tentacle movement under pneumatic actuation in air. The red dotted line traces the bending angle of a long tentacle during the actuation cycle. The red, blue, purple, and orange dots indicate the tip trajectories of the long, medium, short, and super-short tentacles, respectively.

We designed four types of tentacles—Type I super short (1 cm, Ø = 3 mm), Type II short (2 cm, Ø = 5 mm), Type III medium (3 cm, Ø = 5 mm), and Type IV long (4.5 cm, Ø = 5 mm)—as shown in Figure 4a. The super short tentacles are arranged closest to the mouth, forming the inner rings, Ring 1 (r = 14 mm) as shown in Figure 4b. The short, medium, and long tentacles are positioned sequentially from the inner circles to the outermost ring along the surface of the endoderm—forming Ring 2 (r = 23 mm), Ring 3 (r = 28 mm), and Ring 4 (r = 32 mm). In the digital model, each ring contains 12 tentacles evenly spaced around the circumference. The center-to-center distance between adjacent tentacles was determined by the radius of each ring as shown in Table 1. For example, the distance between each Type IV tentacle on Ring 4 is 16.8 mm, measured from center to center. This configuration ensures a balanced and radially symmetric distribution, reflecting the natural arrangement of tentacles in sea anemones and contributing to Soromone’s biomimetic morphology. It is worth noting that the tentacle spacing in the fabricated model may deviate by 1–2 mm from the digital design due to manual fabrication tolerances.

#### 2.3.2. The Manufacturing of Soromone

We used Rhinoceros 3D version 7 software (Robert McNeel & Associates Inc., Seattle, WA, USA) to create the digital design of the Soromone. Based on the complete digital model, customized molds were developed for each element and fabricated using a desktop 3D printer. The soft body was produced through two-part casting techniques. The overall process consists of several steps, as described below.

**Figure 5:**
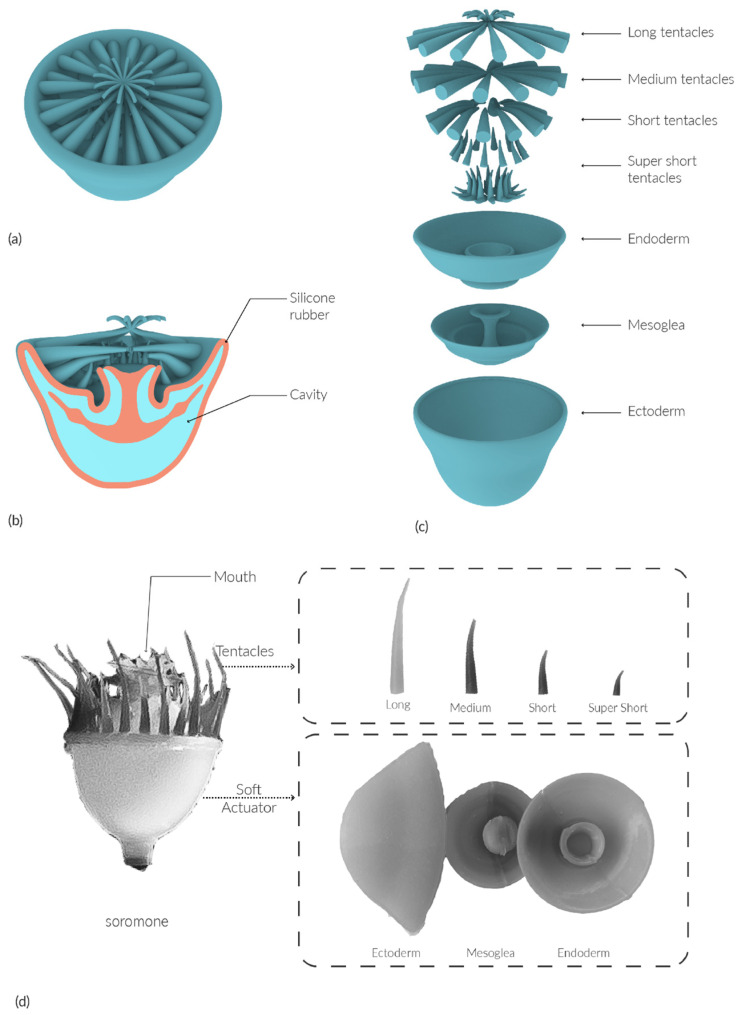
Digital model and real product of a Soromone: (**a**) a digital model of Soromone in static state; (**b**) a 3D section of a digital model of Soromone shows the internal structure; (**c**) a 3D exploded-view of the elements of a Soromone; (**d**) elements of a real product of Soromone.

The first step in the manufacturing of a Soromone is digital modeling. We selected Rhinoceros 3D because it is built on NURBS geometry (Non-Uniform Rational B-Splines), which allows for precise modeling of complex freeform surfaces and curves. This capability is particularly useful for organic, bio-inspired geometries like tentacles, or morphing membranes. Figure 5 illustrates all the elements of a Soromone in digital modeling.

In soft robotics fabrication, the mold casting method provides a fast, low cost, modular, and scalable approach. Although both the body and tentacles use the molding techniques, their distinct processes differ slightly. The soft body (actuator) was produced with a customized two-part mold casting method, whereas the tentacles were fabricated using a customized compression mold casting method.

The two-part mold casting method involves casting one half of a mold followed by the other. Because the soft body contains a volumetric cavity, this method provides an effective way to create the void. To implement it, the mold was digitally divided into two halves and then fabricated using a fused deposition modeling (FDM) 3D printer (CREALITY, ENDER-3 V2, Shenzhen, China).

**Figure 6:**
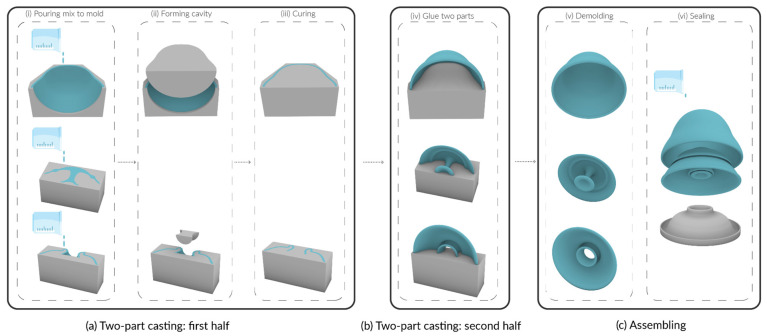
Fabrication steps of the soft actuator: (**a**) first half fabrication; (**b**) second half fabrication; (**c**) assembly.

The fabrication and assembly process of the soft actuator is illustrated in Figure 6. First, we fabricated the first half of the body element (endoderm, mesoglea, or ectoderm). The elastomer was prepared at a 1:1 ratio, poured into the mold, and immediately pressed with a 3D-printed insert to form the cavity. The material was then cured at room temperature (25 °C) for 40 min (Figure 6a). Next, after demolding the cured element, the same procedure was repeated to produce the second half. The two halves were aligned, and the first was glued to the top of the second (Figure 6b). In the final assembly step, a customized support base was used to hold the three elements together during curing. A small amount of mixed material was applied along the edges of the main body (between the endoderm and ectoderm) and around the mouth region (between the ectoderm and mesoglea). After curing, the body of the Soromone was completed.

**Figure 7:**
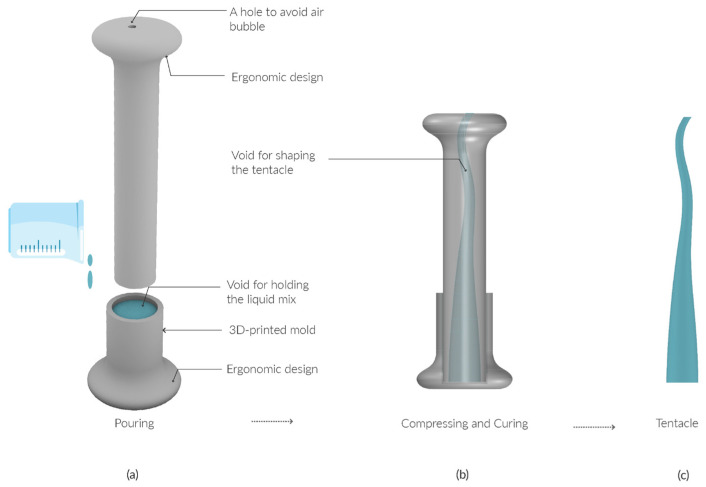
Fabrication steps of tentacles. (**a**) Pouring the mix to the tentacle mold base; (**b**) Compressing the tentacle mold cap into the base and curing; (**c**) Taking out the tentacle from the mold.

The tentacles were also fabricated using mold casting. As discussed in Section 2.2.3, air bubbles are a common issue in this process. To mitigate this, instead of pouring the material directly into a sealed mold, a vent hole was intentionally added to allow trapped air to escape. In addition, a base mold was created to streamline the fabrication process. Because air is lighter than the elastomer, the material was first poured into the base, and the tentacle mold was then pressed down from above. Figure 7 illustrates the long tentacle mold design, which follows the same design principles as the other tentacles. Notably, both the base and the cap include a doorhandle-like knob designed for ergonomic handling. The fabrication steps are shown in Figure 7: (1) pour the mixed elastomer into the base; (2) press the tentacle mold into the base mold; (3) after curing at room temperature (25 °C) for 40 min, demold the tentacle by pulling the two knobs in opposite directions.

To attach the tentacles to the soft actuator, we first glued the innermost ring of short tentacles, as illustrated in Figure 4b, and then proceeded outward to the longer tentacles. After all tentacles were secured, a customized connector was attached to the ectoderm of the soft actuator, which connects to a 2-prong barbed fitting (Adafruit Industries, Brooklyn, NY, USA) for integration with the fluid control system. With this final step, the fabrication of the Soromone was completed.

## 3. Results

### 3.1. Characteristics of Soromone

Figure 5d shows the completed Soromone fabricated with Dragon Skin FX Pro. The wall thickness of both the ectoderm and endoderm are 2 mm. The weights of each element were measured using a professional-grade scale (Acaia Lunar scale, Alhambra, CA, USA). As summarized in Table 2, the ectoderm, mesoglea, and endoderm weigh 20 g, 7.5 g, and 10 g, respectively. The weight percentage parameters could be useful for comparison with those of biological sea anemones. The thickness parameter will serve as a guideline for future manufacturing. Surface area measurements will be essential for determining the distribution of force and pressure during actuation. And the mass provides an estimation of material requirements in future fabrication. The internal cavity was calculated in the digital modeling software as listed in Table 3. The volumetric cavity undergoes the most significant transformation, expanding from 37.5 cm^3^ to 97.5 cm^3^—an increase of 160%.

### 3.2. Morphing Performance and Behavior

A series of experiments were conducted to evaluate the performance of the Soromone. First, we observed and documented the opening and closing of the mouth from a front view. Second, we compared the Soromone’s performance under manual pneumatic and hydraulic control. Third, we examined the tentacle movements under black light. Finally, we tested its ability to push perlite under water.

#### 3.2.1. Electronic Pneumatic Actuation

We used a commercial camera (Fujifilm, Tokyo, Japan) to document the morphing performance. Figure 4c illustrates the overall morphing behavior of a Soromone. The soft actuator was connected to solenoid valves controlled by an Adafruit Circuit Playground Express, with two DC mini air pumps (operating at 4.5 V) serving as the pneumatic power source (Figure 1c). Inflation of the Soromone required 2.5 s, while deflation took 2.2 s. Figure 1d shows a full cycle of pneumatic actuation by the computer control system. Figure 4c provides a closer view focused on the mouth region of the Soromone.

#### 3.2.2. Pneumatic vs. Hydraulic

**Figure 8:**
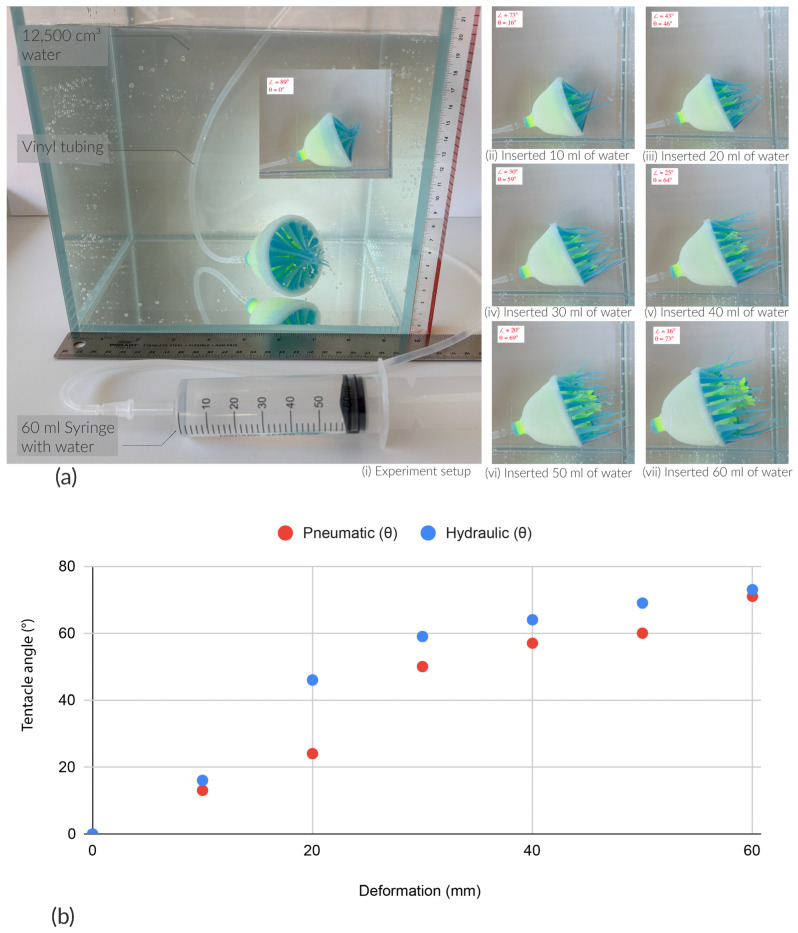
Pneumatic vs. Hydraulic experiments. (**a**) Morphing performance test of a Soromone under water. The red dotted line traces the bending angle of a long tentacle during the actuation cycle. (**b**) A comparison of tentacle bending angles under pneumatic and hydraulic actuations.

**Figure 9:**
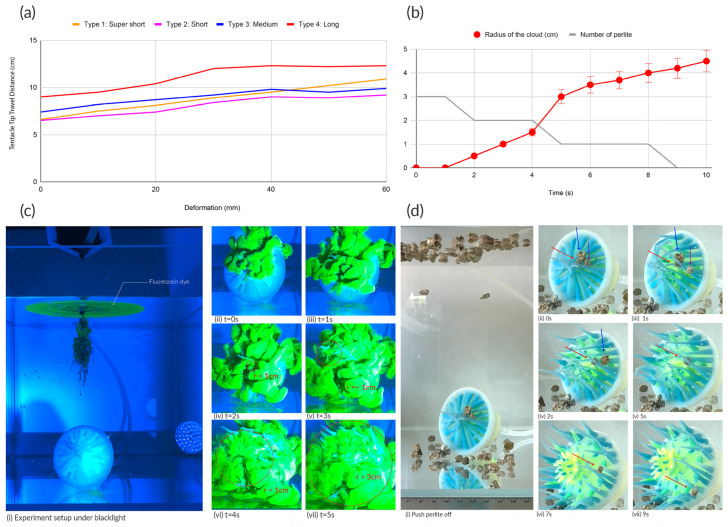
Performance of Soromone. (**a**) Comparing the travel distance of the four types of tentacles from the side views under pneumatic actuation in air; (**b**) Comparison between the size of the dyed cloud and the number of perlite particles attached to the tentacles; (**c**) Study of tentacle-induced movement of the surrounding fluid (**d**) Studying the ability of pushing off perlite in a static water tank. The purple, blue, and red arrows indicate the positions of three perlite particles and their locations throughout the actuation cycle.

We observed and recorded both the pneumatic and hydraulic tests using an iPhone 16 (Cupertino, CA, USA). Figure 4d and Figure 8a illustrate the experimental setup and results. For the pneumatic test, a 60 mL syringe was connected to the Soromone via vinyl tubing. The Soromone was positioned in a side view on a mat to measure tentacles’ movement. Photographs documented the Soromone’s morphing states as air was injected from 0 mL to 60 mL, in 10 mL increments. Figure 4d illustrates the angle of a tentacle change from 0 degrees to 71 degrees by inserting 60 mL of air. Additionally, each type of tentacle’s tip is annotated as orange (Type I), blue (Type II), pink (Type III), and red (Type IV). Figure 9a illustrates the travel trajectories of the four types of tentacles under pneumatic actuation in air. For example, Type I Super short tentacle moves from 6.6 cm to 10.9 cm, and Type 4 Long tentacle moves from 9 cm to 12.3 cm. The more consistent and gradual extension observed in Type I (orange line in Figure 9a), relative to Type II and Type III, is likely influenced by the geometry of the mouth design, particularly the mesoglea layer.

For the hydraulic test, the same 60 mL syringe was used. The Soromone was prefilled with tap water, and then additional water was injected from 0 mL to 60 mL, again in 10 mL increments. The water tank measured 25 cm × 25 cm × 25 cm, and the tap water was filled to a height of 20 cm (12,500 cm^3^). Comparing the two actuation modes, Soromone’s mouth exhibited greater stretching under hydraulic actuation, particularly from 40 mL onward, indicating that hydraulic control is more effective (as shown in Figure 9b). As a side note, a Soromone can also operate underwater using pneumatic actuation; however, because the body is filled with air, it will float unless anchored and therefore, require high pressure pneumatic pumps.

Figure 3 presents a digital illustration of the internal morphing mechanism of a Soromone during unactuated and actuated states. As observed in the pneumatic and hydraulic tests, the ectoderm maintains its bell-shaped form with minimal deformation under normal actuation. In contrast, the endoderm undergoes more dramatic morphological changes in both hydraulic and pneumatic tests: its inward curves push outward, moving the mouth from within the body to the surface. Although its shape changes, the surface area of the endoderm remains nearly constant, as it simply flips from one orientation to another. The mesoglea is not directly visible in the current experiments; instead, its morphing behavior is schematically represented in Figure 3. The mesoglea has a mushroom-like form: the umbrella edge consists of relatively thinner material that bends upward with ease, while the central region is much thicker and curves downward into the stem. This structure provides both support and restoring force, enabling the endoderm to return organically to its original shape.

#### 3.2.3. Task Experiment

To further investigate the mechanism and movement of a Soromone, we conducted two experiments: (1) tentacle-induced movement of the surrounding fluid and (2) the ability to push off perlite in a static water tank. The key findings are summarized as follows: (1) tentacle movement gently increases fluid motion in the immediate surroundings, and (2) the opening and closing of the mouth can push lightweight objects away from its surface.

Figure 9c illustrates the first experiment, in which a Soromone was prefilled with tap water in its cavity and then placed inside the water tank. The fluorescein dye was prepared by mixing fluorescein sodium (Carolina Biological Supply Company, Burlington, NC, USA) with filtered tap water at a 1:3 ratio. After the dye was dripped into the tank (Figure 9c(i)), the Soromone was actuated. From 0 to 1 s, no tentacle-induced movement was observed. At 2 s, the tentacles began to move, creating a localized cloudy effect (circled in red in Figure 9c(iv–vii)). Between 4 and 5 s, this cloudy effect spread around the Soromone as the tentacles continued moving. Similar to natural sea anemone tentacles, the Soromone’s tentacles did not generate strong water currents but instead exhibited gentle wiggling and bending in the direction of the surrounding flow.

Figure 9d illustrates the second experiment in which a Soromone was also prefilled with water. Perlite, a lightweight natural glass in pebble form, was used to test whether the Soromone could push off small objects. A total of 100 perlite pieces were prepared, ranging in size from 3 mm to 8 mm, with a combined weight of 13.3 g. After all of the perlite was released into the water, some pieces floated while others sank. Three perlite pieces settled on the Soromone. When the Soromone was actuated, all three pieces fell off after the first actuation cycle. Figure 9d(i–vii) illustrate this process.

These two experiments demonstrate an inverse relationship between the size of the dyed cloud and the number of perlite particles attached to the tentacles, as shown in Figure 9b. Both effects result from the actuation of Soromone. During each actuation cycle, the tentacle movements increase the size of the dyed cloud while simultaneously dislodging nearby perlite particles.

One of the advantages of Soromone is the structure maintains performance consistency across repeated actuation cycles, both in air and underwater. Additionally, the organic morphology and motion enhance realism of the robot performance, making Soromone suitable for underwater interaction with marine animals.

## 4. Discussion and Conclusions

This paper introduces a soft tectonics design methodology for soft robotics based on the architectural theory of tectonics. Most previously existing pneumatic actuators are fabricated with two layers, in which the external layer is expandable, and the inner layer is inextensible [34]. Such designs face clear limitations, highlighting the urgent need for new methodologies. Drawing inspiration from tectonic theory in architecture, we propose soft robotics-based soft tectonics as a novel design and manufacturing approach.

To demonstrate this design framework, we presented the Soromone, a cnidarian-inspired soft underwater robot capable of performing sea-anemone-like feeding movements. By studying cnidarians—particularly jellyfish and sea anemones—we developed a soft robot that functions both in air and underwater, embodying biologically expressive morphing behaviors. The performance was evaluated through pneumatic and hydraulic lab tests. The robot successfully demonstrates sea anemone-like tentacle movements.

**Figure 10:**
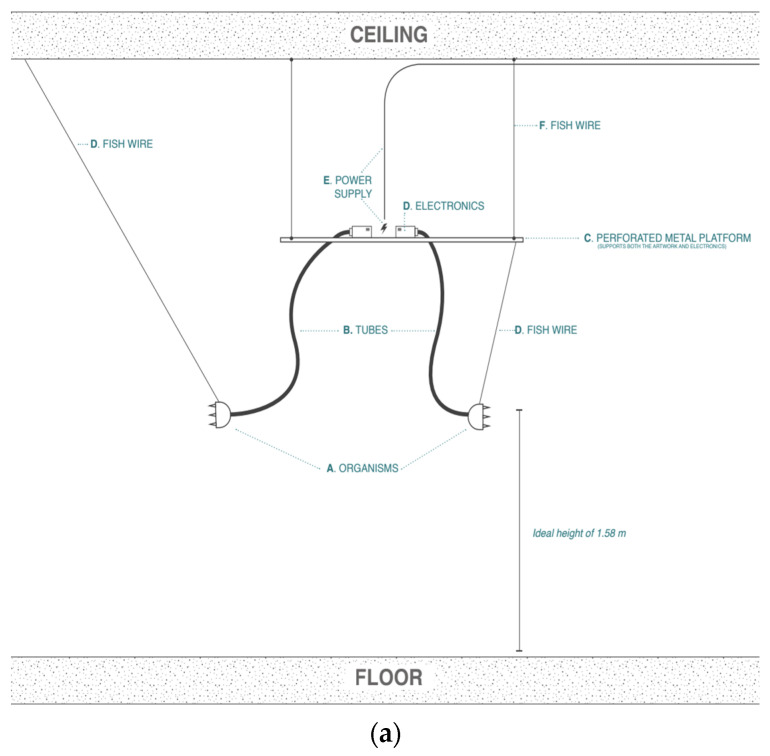

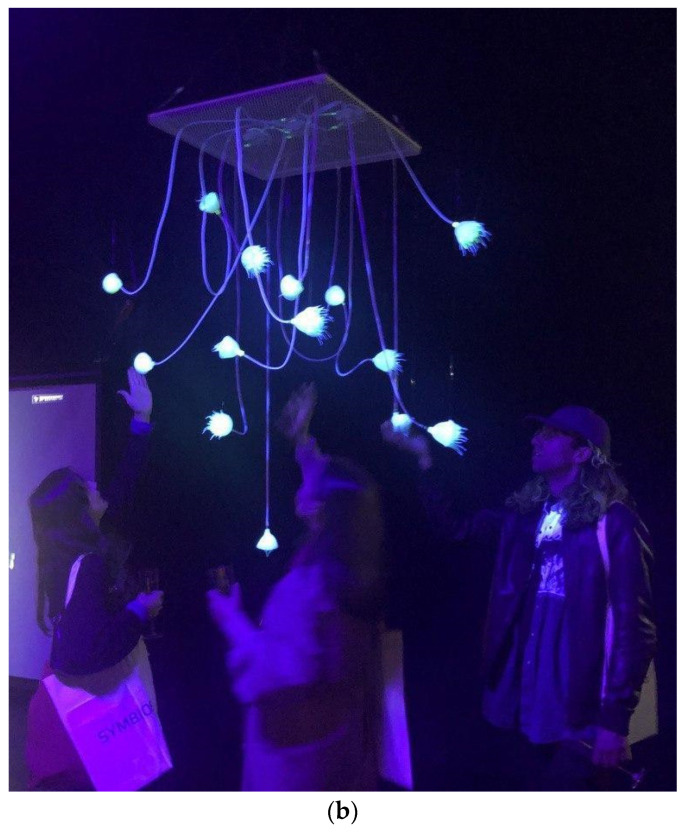
The Soromone shown in a public exhibition. (**a**) A Soromone system set up in a public space; (**b**) Visitors’ interaction with the Soromone.

A key advantage of the Soromone is its durability. Unlike conventional pneumatic soft robots, whose materials degrade over repeated actuation cycles, the Soromone’s structural design minimizes material fatigue. As highlighted in the introduction, the Soromone was developed for public exhibition as part of advancing the field of ethorobotics. Installed in a gallery setting for public engagement (Figure 10), the robot successfully operated continuously for a week-long period (≈4.11 × 10^4^ cycles) under a duty cycle of 14.7 s per cycle (2.5 s inflate → 5 s hold → 2.2 s deflate → 5 s hold) without detectable loss of morphing amplitude. As long as the hardware components (e.g., pumps) remain functional, the robot can operate over extended periods, offering a much longer lifespan than typical elastomer-based soft robots. Table 4 compares sea anemone–inspired soft robots across actuator fabrication method, materials, tentacle topology (number of types/rings), test environment, performance, and reported durability from the literature. Most prior works evaluated in air (one in water) and did not report life cycle data (durability = N/A). These results indicate that the soft-tectonics method delivers realistic biomimicry morphology and mitigates common fatigue in elastomeric actuators.

Another advantage of the Soromone is its realism compared to other soft robots. This opens the door to a variety of potential applications, including underwater animal–robot interaction, aquarium display, and long-term observation of marine organisms.

For future work, we plan to develop a large-scale version of the Soromone capable of untethered operation by integrating sensors, control systems, and power sources into the design. In addition, exploring the Soromone as a platform for ethorobotics will provide valuable opportunities for advancing both scientific understanding and public engagement.

## 5. Patents

The soft tectonics design method of the soft robot, Soromone, has been disclosed to the San Diego State University Technology Transfer Office and is filed at the United States Patent and Trademark Office (USPTO) under application number 63/875,219.

## Figures and Tables

**Table 1:** Geometric parameters of tentacle arrangement in Soromone digital model.

Ring	Radius (mm)	Tentacle Type	Length (mm)	Distance Between Tentacles (mm)
Ring 1	14	Type I Super short	10	7.3
Ring 2	23	Type II Short	20	12
Ring 3	28	Type III Medium	30	14.7
Ring 4	32	Type IV Long	45	16.8

**Table 2:** Specification of the Soft Actuator.

Name of the Elements	Weight	Weight Percentage	Thickness	Surface Area	Mass
Ectoderm	20 g	~39%	2 mm	72 cm^2^	15.6 cm^3^
Mesoglea	7.5 g	~15%	Varied	58 cm^2^	7.2 cm^3^
Endodern	10.5 g	~ 20%	2 mm	50 cm^2^	9.9 cm^3^
Tentacles	~12 g	~24%	Varied	Varied	Varied
Soromone	51 g	100%	-	-	-

**Table 3:** A comparison of Mouth Closed and Mouth Open of the Soft Actuator.

States	Ectoderm	Mesoglea	Endodern	Cavity
Closed	72 cm^2^	58 cm^2^	50 cm^2^	37.5 cm^3^
Open	80 cm^2^	58 cm^2^	52 cm^2^	97.5 cm^3^

**Table 4:** A comparison of sea anemone-inspired soft robots presented in the literature.

Project (Year)	Actuator Fabrication Method	Material	Number of Tentacle Types	Number of Tentacle Rings	Performance	Test Environment	Reported Durability
Bionic torus (2020) [19]	Double-layered cylindrical tube of a torus	Thermoplastic-rubber with sealed liquid	N/A	N/A	Soft torus gripping mimics the hunting process of a sea anemone	In air	N/A
RetracTip (2022) [20]	An array of rigid pins distributed on a spherical membrane	3D printed rigid pins as tentacles, bistable dome membranes	1	4	Tentacles and retract muscle gripping mimics sea anemones- preying behavior	In air	N/A
Magnetic soft robot (2021) [21]	Four tentacles on a hollow body	Magnetic NdFeB/Ecoflex composites	1	1	Tentacles sense water flow, and the hollow body mimics a sea anemone’s shrinkable body.	In water	N/A
Continuum arm (2021) [22]	SMA tendon wire	Silicone, wire, spring, photosensitive resin 3D-printed skeleton	1	1 (r = 60 mm)	Tentacles gripping	In air	N/A
Soromone (2025) [this paper]	Soft tectonics (three layers of membranes with 60 tentacles)	Silicone	4	4 (see Table 1)	Soromone’s mouth opening/closing mimic sea anemone’s tentacle wiggling behavior and feeding movement	In air and water	≥4.11 × 10^4^ cycles

## Data Availability

Data is contained within the article.

## References

[1] Butler D. (2016). A world where everyone has a robot: Why 2040 could blow your mind. Nature.

[2] Bao G., Fang H., Chen L., Wan Y., Xu F., Yang Q., Zhang L. (2018). Soft robotics: Academic insights and perspectives through bibliometric analysis. Soft Robot..

[3] Wada K., Shibata T. (2007). Living with Seal Robots—Its Sociopsychological and Physiological Influences on the Elderly at a Care House. IEEE Trans. Robot..

[4] Webster R.J., Okamura A.M., Cowan N.J., Taylor R.H. (2012). An Active Cannula for Bio-Sensing and Surgical Intervention. U.S. Patent.

[5] Beatty R., Mendez K.L., Schreiber L.H.J., Tarpey R., Whyte W., Fan Y., Robinson S.T., O’Dwyer J., Simpkin A.J., Tannian J. (2023). Soft robot–mediated autonomous adaptation to fibrotic capsule formation for improved drug delivery. Sci. Robot..

[6] Arfaee M., Vis A., Bartels P.A.A., van Laake L.C., Lorenzon L., Ibrahim D.M., Zrinscak D., Smits A.I.P.M., Henseler A., Cianchetti M. (2025). A soft robotic total artificial hybrid heart. Nat. Commun..

[7] Galloway K.C., Becker K.P., Phillips B., Kirby J., Licht S., Tchernov D., Wood R.J., Gruber D.F. (2016). Soft robotic grippers for biological sampling on deep reefs. Soft Robot..

[8] Yang G.-Z., Bellingham J., Dupont P.E., Fischer P., Floridi L., Full R., Jacobstein N., Kumar V., McNutt M., Merrifield R. (2018). The grand challenges of science robotics. Sci. Robot..

[9] Aracri S., Giorgio-Serchi F., Suaria G., Sayed M.E., Nemitz M.P., Mahon S., Stokes A.A. (2021). Soft robots for ocean exploration and offshore operations: A perspective. Soft Robot..

[10] Katzschmann R.K., DelPreto J., MacCurdy R., Rus D. (2018). Exploration of underwater life with an acoustically controlled soft robotic fish. Sci. Robot..

[11] Brooker R.M., Feeney W.E., Sih T.L., Ferrari M.C.O., Chivers D.P. (2019). Comparative diversity of anemone-associated fishes and decapod crustaceans in a Belizean coral reef and seagrass system. Mar. Biodivers..

[12] Levy N., Marques J.A., Simon-Blecher N., Bourne D.G., Doniger T., Benichou J.I.C., Lim J.Y., Tarazi E., Levy O. (2024). Ecosystem transplant from a healthy reef boosts coral health at a degraded reef. Nat. Commun..

[13] Baumgarten S., Simakov O., Esherick L.Y., Liew Y.J., Lehnert E.M., Michell C.T., Li Y., Hambleton E.A., Guse A., Oates M.E. (2015). The genome of *Aiptasia*, a sea anemone model for coral symbiosis. Proc. Natl. Acad. Sci. USA.

[14] Wang T., Joo H.-J., Song S., Hu W., Keplinger C., Sitti M. (2023). A versatile jellyfish-like robotic platform for effective underwater propulsion and manipulation. Sci. Adv..

[15] Wu M., Afridi W.H., Wu J., Afridi R.H., Wang K., Zheng X., Wang C., Xie G. (2024). Octopus-Inspired underwater soft robotic gripper with crawling and swimming capabilities. Research.

[16] Fras J., Noh Y., Macias M., Wurdemann H., Althoefer K. Bio-inspired octopus robot based on novel soft fluidic actuator. Proceedings of the IEEE International Conference on Robotics and Automation (ICRA).

[17] Ren Z., Hu W., Dong X., Sitti M. (2019). Multi-functional soft-bodied jellyfish-like swimming. Nat. Commun..

[18] Lee J., Yoon Y., Park H., Choi J., Jung Y., Ko S.H., Yeo W.-H. (2022). Bioinspired soft robotic fish for Wireless underwater control of gliding locomotion. Adv. Intell. Syst..

[19] Zang H., Liao B., Lang X., Zhao Z.-L., Yuan W., Feng X.-Q. (2020). Bionic torus as a self-adaptive soft grasper in robots. Appl. Phys. Lett..

[20] Qi Q., Xiang C., Ho V.A., Rossiter J. (2022). A sea-anemone-inspired, multifunctional, bistable gripper. Soft Robot..

[21] Wang Q., Wu Z., Huang J., Du Z., Yue Y., Chen D., Li D., Su B. (2021). Integration of sensing and shape-deforming capabilities for a bioinspired soft robot. Compos. Part B Eng..

[22] Yang J., Ren C., Yang C., Wang Y., Wan S., Kang R. (2021). Design of a flexible capture mechanism inspired by sea anemone for non-cooperative targets. Chin. J. Mech. Eng..

[23] Youssef S.M., Soliman M., Saleh M.A., Mousa M.A., Elsamanty M., Radwan A.G. (2022). Underwater Soft Robotics: A Review of Bioinspiration in Design, Actuation, Modeling, and Control. Micromachines.

[24] Abdai J., Miklósi A. (2025). An Introduction to Ethorobotics: Robotics and the Study of Animal Behaviour.

[25] Marchese A.D., Katzschmann R., Rus D.L. (2015). A recipe for soft fluidic elastomer robots. Soft Robot..

[26] Xavier M.S., Tawk C.D., Zolfagharian A., Pinskier J., Howard D., Young T., Lai J., Harrison S.M., Yong Y.K., Bodaghi M. (2022). Soft pneumatic actuators: A review of design, fabrication, modeling, sensing, control and applications. IEEE Access.

[27] Yordanova N. (2019). A new approach to the concept of tectonics. Structures and Architecture-Bridging the Gap and Crossing Borders.

[28] Thomsen M.R., Bech K., Tamke M., Glynn R., Sheil B. (2011). THAW: Imagining a Soft Tectonics. Fabricate: Making Digital Architecture.

[29] Dong X., Luo X., Zhao H., Qiao C., Li J., Yi J., Yang L., Oropeza F.J., Hu T.S., Xu Q. (2022). Recent advances in biomimetic soft robotics: Fabrication approaches, driven strategies and applications. Soft Matter.

[30] Howell L.L., Olsen B.M., Magleby S.P. (2013). Part Three Synthesis Of Compliant Mechanisms. Handbook of Compliant Mechanisms.

[31] OpenStax (2022). Phylum Cnidaria (Section 5.8.3). General Biology 2e. Biology LibreTexts. https://bio.libretexts.org/Bookshelves/Introductory_and_General_Biology/General_Biology_2e_(OpenStax)/05:_Unit_V-_Biological_Diversity/5.08:_Invertebrates/5.8.03:_Phylum_Cnidaria.

[32] Ames C.L. (2018). Medusa: A review of an ancient cnidarian body form. Marine Organisms as Model Systems in Biology and Medicine.

[33] Ikmi A., Steenbergen P.J., Anzo M., McMullen M.R., Stokkermans A., Ellington L.R., Gibson M.C. (2020). Feeding-dependent tentacle development in the sea anemone *Nematostella vectensis*. Nat. Commun..

[34] Su H., Hou X., Zhang X., Qi W., Cai S., Xiong X., Guo J. (2022). Pneumatic Soft Robots: Challenges and Benefits. Actuators.

